# Damage evolution of Cu-inductors used for electromagnetic forming

**DOI:** 10.1038/s41598-025-14135-4

**Published:** 2025-08-04

**Authors:** Lisa-Marie Rymer, Lisa Winter, Maik Linnemann, Sven Winter, Verena Psyk, Thomas Lampke

**Affiliations:** 1 Institute of Materials Science and Engineering, Materials and Surface Engineering Group, Chemnitz University of Technology, 09107 Chemnitz, Germany; 2 Fraunhofer Institute of Machine Tools and Forming Technology IWU, Reichenhainer Straße 88, 09126 Chemnitz, Germany

**Keywords:** Electromagnetic forming (EMF), Copper alloy, Microstructure, Electron backscatter diffraction (EBSD), Materials science, Mechanical engineering

## Abstract

Electromagnetic forming (EMF) is a high-speed forming technology using the interactions of pulsed currents and magnetic fields to apply Lorentz forces to electrically conductive workpieces. The damage behavior of Cu-inductors used for EMF was investigated by electron microscopy, particularly electron backscatter diffraction (EBSD) and energy dispersive x-ray spectroscopy (EDS). The process-specific electrical-thermo-mechanical load leads to plastic deformations on the inductor and melting and re-solidification of grain boundaries. Both weaken the inductor material. Cracks propagate at grain boundaries, where the thermo-mechanical load is concentrated, and become larger after each discharge. As a result, blowholes form, which cause failure of the inductor. Annealing and recrystallization processes as well as local melting at grain boundaries and formation of blowholes due to joule heating are probably the origin of the damage evolution during EMF. Understanding the correlations of these microstructural mechanisms will enable targeted heat treatment for wear-resistant inductors in the future.

## Introduction

**Fig. 1:**
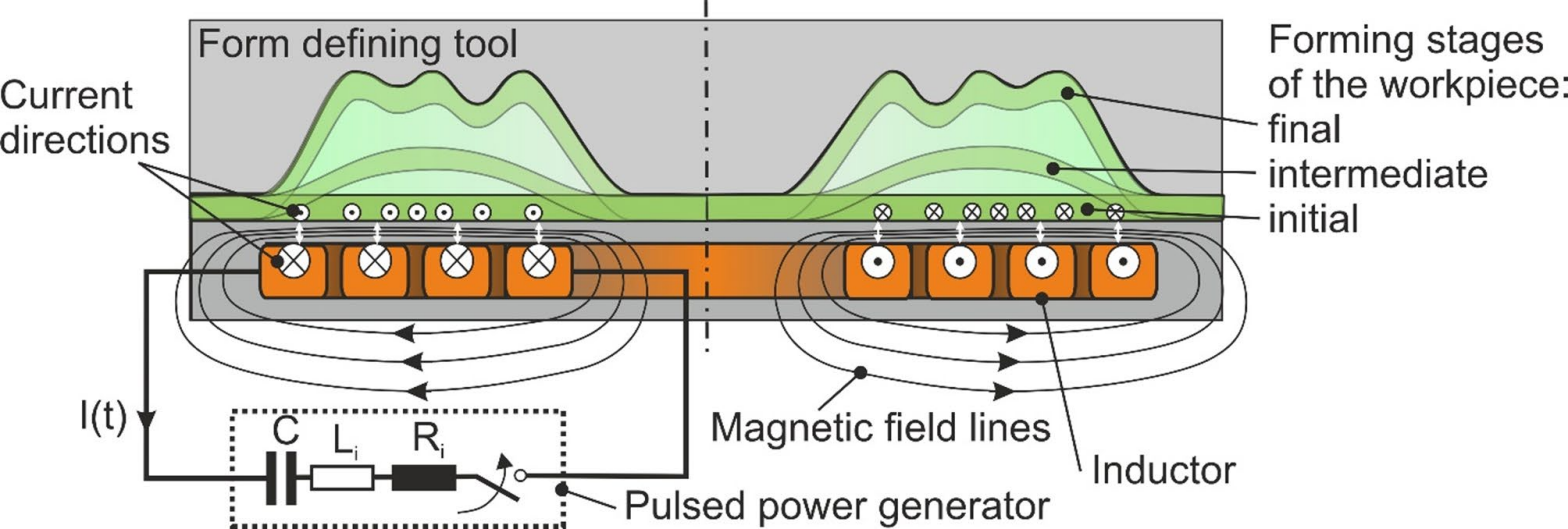
Set-up of EMF. The set-up of EMF consists of the pulsed power generator (forming machine), the inductor (active tool) and the workpiece, which is positioned close to but without contacting the inductor. In addition, different forming stages are shown from the initial to the final stage.

Electromagnetic forming (EMF) enables the shaping, cutting, or joining of tube and sheet metal workpieces made of (highly) conductive materials such as aluminum, copper, and their alloys, as well as mild steel^1^. The process-specific high-rate deformation enables high strains^2^ and complex geometries without cracks^3^. Moreover, springback is minimal compared to conventional forming processes^4^. As shown in Fig. 1, the set-up of EMF consists of the pulsed power generator (forming machine), the inductor (active tool), the workpiece, which is positioned close to but without contacting the inductor, and, depending on the specific application, a form-defining or cutting tool and the joining partner, respectively.

For the process, the capacitor battery, which is part of the forming machine, is discharged via the inductor, resulting in a damped sinusoidal inductor current with amplitudes ranging from several 10 kA to several 100 kA and a corresponding magnetic field, which induces additional eddy currents in the electrically conductive workpiece. The interactions of the magnetic field and the currents cause Lorentz forces, which can be mathematically transformed to surface forces, the so-called magnetic pressure. This magnetic pressure can exhibit maximum values of several hundred MPa with pulse durations ranging from 15 µs to 100 µs. If it reaches the flow stress of the workpiece, the material is plastically deformed at strain rates of up to 10^5^ s^-1^^5^.

In process and tool design, it must be considered that, corresponding to Newton’s third axiom, the high loads required for the workpiece deformation also affect the tool. In this context, in addition to the mechanical stresses, also the electrical loads, and especially in the case of series production, the thermal loads have to be considered. Depending on the manufacturing task and the process and tool design, the constantly repeated combination of the electrical-thermo-mechanical load can cause progressing damage to the inductor. In the literature, the stress state around a pre-existing crack tip, which was induced by an electric current that causes a self-induced magnetic field, was numerically evaluated^6^. A stress singularity was found around the crack tip. However, this phenomenon also occurs at non-crack surfaces, when a high electrical-thermo-mechanical load, e.g., by EMF, is applied, leading to the initiation of cracks at the surface. Scheffler^7^ assumed that high current densities lead to large Lorentz forces, which are the origin of crack initiation. Similar damage behavior was observed in a fieldshaper made of a CuNiSi alloy that was used during EMF^8^. In their study, the first crack was initiated at the position where the highest stress level was reached. The large stress development was caused by the radial component of the Lorentz force. In addition, not only the fieldshaper is damaged, but also the inductor, as mentioned earlier. Due to proximity effects, the electrical current and thus also the inductor loading are obviously concentrated at the surface close to the workpiece material, but the details of inductor damage and failure, respectively, have not been fully understood yet. Furthermore, the formation of cracks is critical for an inductor’s lifetime, as it cannot be used for further discharges and will damage the workpiece as well. Therefore, this study deals with the influence of microstructural characteristics such as grain and twin boundaries as well as particles in order to understand the damage behavior and evolution of Cu-inductors used during EMF.

## Methods

**Fig. 2:**
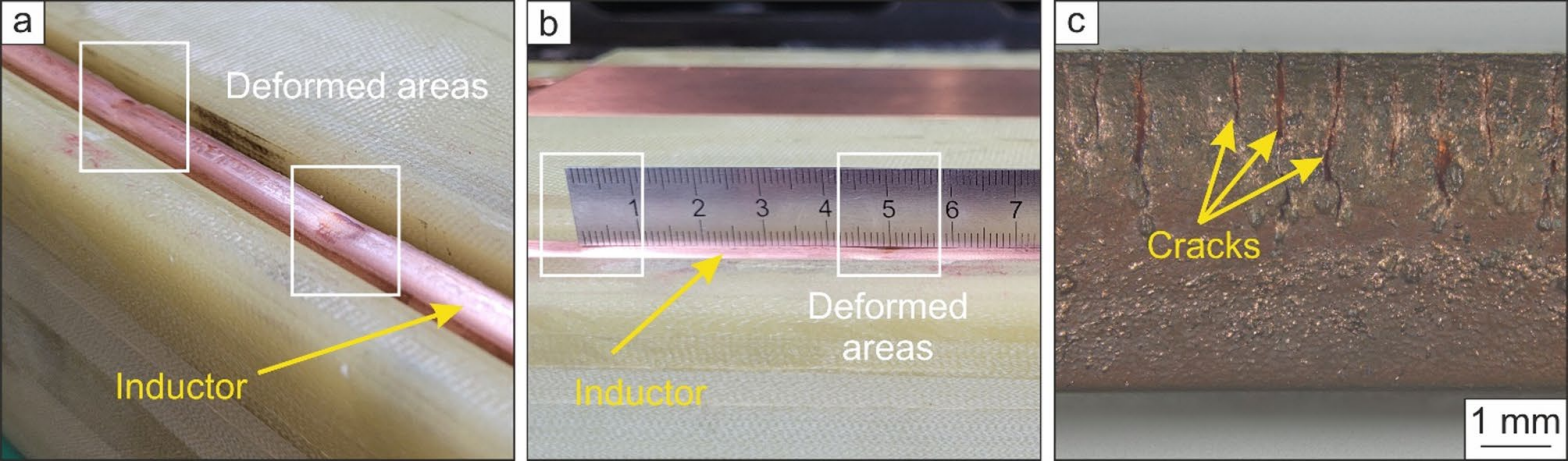
Macroscopic damage behavior of Cu-inductors after EMF. Deformed areas on the inductor are present after use on the side close to the workpiece material (**a**). These deformed areas occur with a distance of 50 mm (**b**). Surface cracks have propagated deep into the inductor (**c**).

The investigated inductors are made of a CuCr1Zr (Fig. 2a–b) and a CuBe2 (Fig. 2c) alloy with cross-sections ranging from 60 mm^2^ to 223 mm^2^. The nominal chemical composition is given in Table 1.

**Table 1:** Chemical composition of the alloys CuCr1Zr and CuBe2 in wt.-%.

	Fe	Si	Cr	Co	Ni	Co + Ni	Zr	Be	Others	Cu
CuCr1Zr	0.08	< 0.1	0.5–1.2				0.03–0.3		0.2	rest
CuBe2	< 0.1	< 0.1		< 0.3	< 0.3	0.2–0.5		1.8–2.0	< 0.5	rest

The CuCrZr1 inductor was exposed to approximately 200 discharges with high voltages (> 20 kV) but low current densities (< 1 kA/mm²), while the CuBe2 inductor was only exposed to a few discharges with low voltages (< 15 kV) and high current densities (> 5 kA/mm²). The macroscopic investigations were done by stereo microscopy (Olympus MVX10, Olympus, Japan). After the macroscopic investigations, a cross-section of the CuBe2 inductor from Fig. 2c was cut and embedded in conductive resin. Next, the cross-section was metallographically prepared using SiC papers (up to grit P4000) and polished with 3 μm and 1 μm diamond suspension. The cross-section was finished using vibration polishing with OPS that is combined with a diluted solution of hydrogen peroxide and ammonium hydroxide. The metallographically prepared cross-section was examined using scanning electron microscopy (SEM, NEON 40 EsB, Zeiss, Jena, Germany) with a backscatter electron (BSE) detector at 20 kV and a working distance of about 10 mm. To analyze the formed blowhole in detail, electron backscatter diffraction (EBSD) was performed using the same SEM. An acceleration voltage of 20 kV, a working distance of about 18 mm, and a step size of 2 μm (Fig. 4) and 0.05 μm (Fig. 6) were used. The gained EBSD data were evaluated with the EDAX OIM Analysis 8 software. The data was cleaned using the grain CI standardization (grain tolerance angle 15.0°, minimum grain size 5, multi row required) and the neighbor CI Correlation (minimum CI of 0.1, single iteration). In addition, the chemical composition was also determined by X-ray fluorescence analysis (XRF, M4 Tornado, Bruker-AXS, Karlsruhe, Germany). Furthermore, the element distribution was analyzed by energy-dispersive X-ray spectroscopy (EDS) with the NEON 40 EsB. The acceleration voltage was set to 20 kV and the high-current mode was used. The working distance was set to 6.5 mm.

## Results

### Macroscopic damage behavior of Cu-inductors after electromagnetic forming

The electrical-thermo-mechanical load induced by EMF after several discharges causes damage in a CuCr1Zr inductor (Fig. 2a, b) and a CuBe2 (Fig. 2c) is shown macroscopically in Fig. 2.

Figure 2a–c shows the deformed areas due to induced electrical and mechanical load as well as the temperature-dependent surface damages and cracks after repeated use of the inductor. The deformed areas occur periodically in the CuCr1Zr-inductor with a distance of 50 mm between them (Fig. 2a, b). These plastically deformed areas were present after approximately 200 discharges with high voltages (> 20 kV) but low current densities (< 1 kA/mm²). However, the more critical damage behavior of a Cu-inductor during EMF was found in Fig. 2c. This inductor was made of CuBe2 and exposed to a few discharges with low voltages (< 15 kV) and high current densities (> 5kA/mm²). The cross-section of the inductor shows surface cracks, which extend deeply into the material (Fig. 2c). The cracks are very critical as they indicate that the inductor failed and cannot be used anymore, despite the low number of performed discharges. If such an inductor is further used, damage of the workpiece material will follow. Therefore, the inductor has to be replaced, which is cost-intensive and time-consuming.

### Microscopic damage evolution of Cu-inductors after electromagnetic forming

**Fig. 3:**
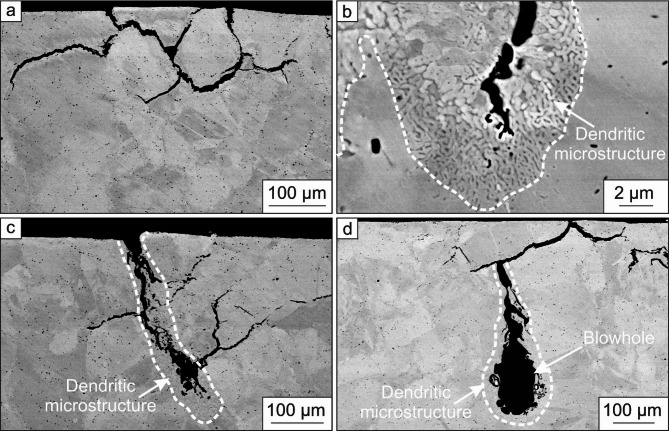
BSE images of the inductor after use. Cracks form at grain boundaries (**a**). At these cracks, a fine dendritic microstructure is present (**b**). The surface damage evolves to pores surrounded by a dendritic microstructure (**c**), developing a blowhole with an increasing number of discharges (**d**).

To clarify the origin of the observed failure behavior, a metallographically prepared cross-section of the CuBe2-inductor in Fig. 2c was investigated using SEM. In Fig. 3, the obtained BSE images are shown.

Noticeable in Fig. 3a, cracks were formed starting from the surface. The cracks propagate predominantly at the grain boundaries. In addition, fine dendrites are surrounding these cracks (Fig. 3b). Such fine dendrites are possibly formed due to local melting and re-solidification processes at the grain boundaries. Furthermore, some of these cracks extend and form larger pores after several discharges (Fig. 3c). The pores are surrounded by a dendritic microstructure as well (white dashed line in Fig. 3c). The final step of damage evolution is the formation of blowholes, which either start from the surface or existing cracks (Fig. 3d). The dendritic microstructure at the blowholes is marked by a white dashed line in Fig. 3d. The previously found cracks in Fig. 2c are now attributed to be blowholes, as their shape and size correspond to the damage characteristics in Fig. 2c.

**Fig. 4:**
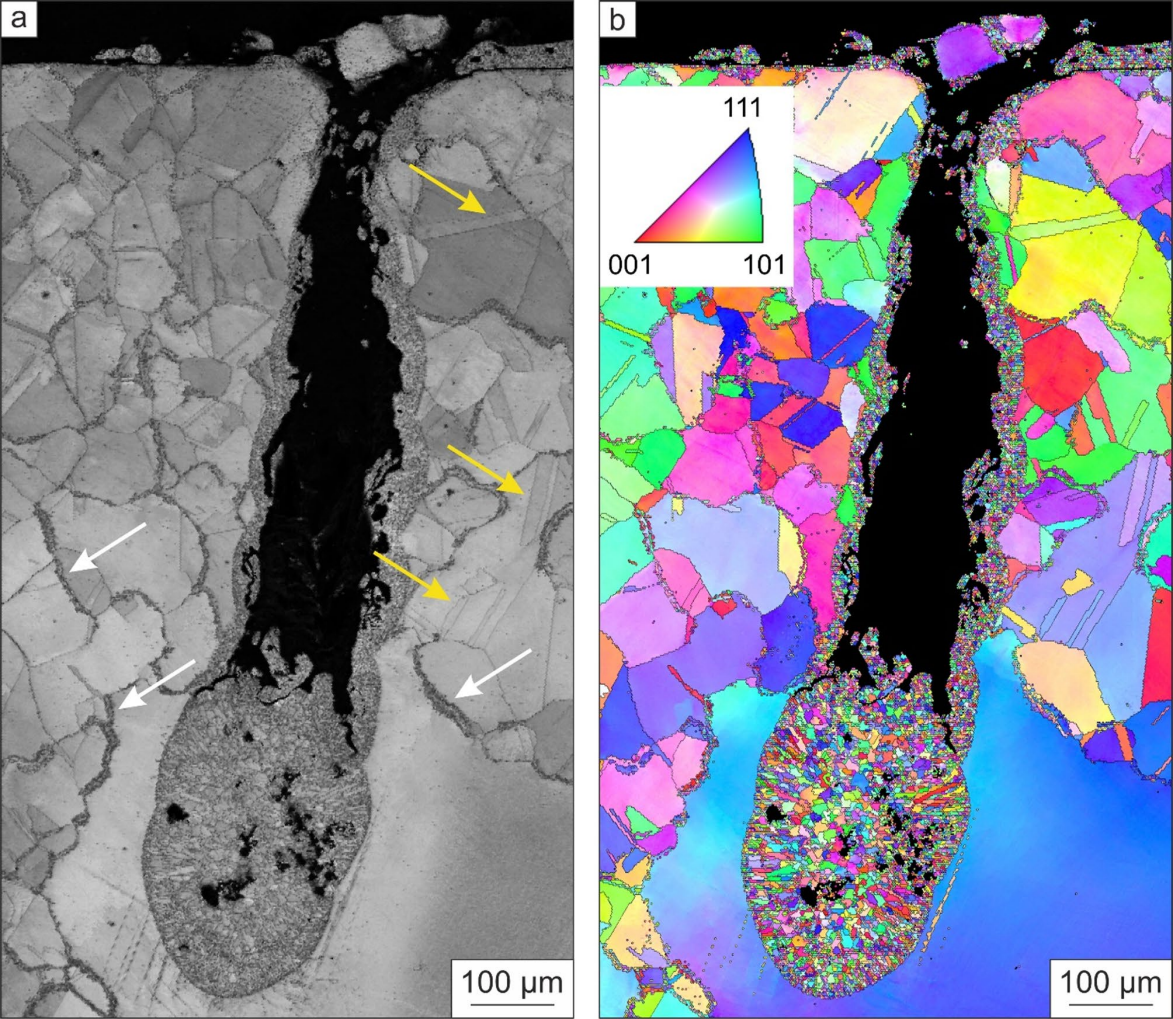
Image quality map (**a**) and orientation map (**b**) of a blowhole. A fine-grained microstructure is present in the blowhole and at the grain boundaries (white arrows). In contrast, such a fine-grained microstructure is not found at twin boundaries (yellow arrows).

To analyze the formed blowhole in detail, EBSD was performed. The obtained image quality (IQ) map (a) and the orientation (inverse pole figure, IPF) map (b) are given in Fig. 4.

EBSD measurements confirm that a fine-grained microstructure in the blowhole and at the grain boundaries. Considering their crystallographic orientation, the grains in the blowhole are randomly distributed. In addition, fine grained material is also found outside the blowhole, which lead to the assumption that material from inside was ejected during EMF. Such a fine-grained microstructure is also present at the grain boundaries (Fig. 4a, white arrows). In contrast to the microstructure at grain boundaries, the grains are not refined at annealing twins (Fig. 4b, yellow arrows).

**Fig. 5:**
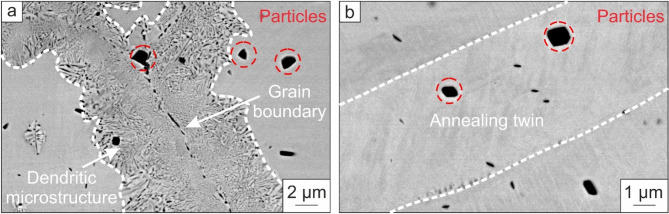
BSE images of a grain boundary (**a**) and an annealing twin (**b**). At the grain boundary, a fine dendritic microstructure is present. In contrast, such a dendritic microstructure was not found at annealing twins.

In Figure 5, higher magnified BSE images of a grain boundary (a) and an annealing twin boundary (b) are shown.

The fine-grained microstructure at the grain boundaries found in the EBSD measurements is dendritic as well. In contrast, no dendritic microstructure appears at the twin boundary (Fig. 5b). In addition, pores form at the grain boundary, and the dendritic microstructure becomes coarser with a larger distance from the grain boundary (Fig. 5a). Furthermore, particles are found in or next to the grain and twin boundaries. To identify the chemical composition of the particles and if other elements are preferably located next to the grain and twin boundaries, XRF and EDS were used.

**Fig. 6:**
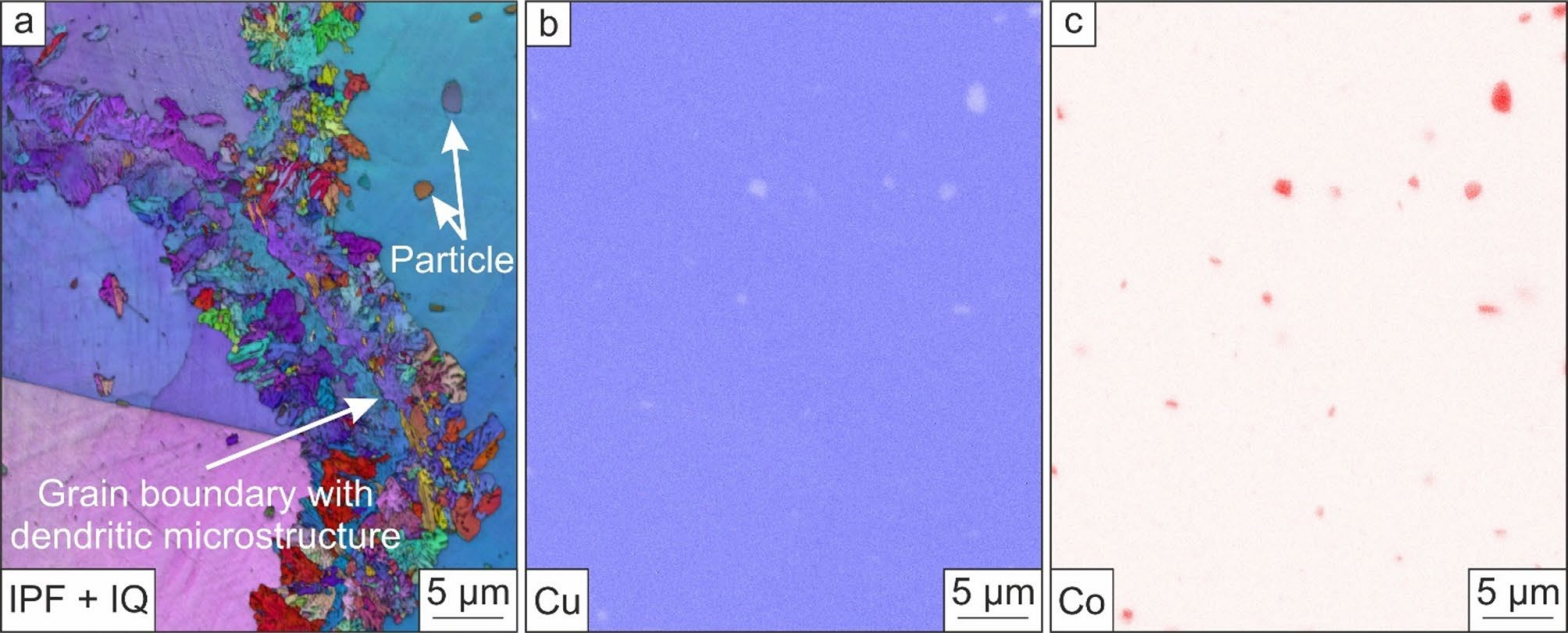
Orientation map of a grain boundary (**a**). To identify the elemental distribution, EDS was used. It was found that Cu is randomly distributed (**b**). In addition, Co-rich particles were found, which are also randomly distributed.

As shown in Fig. 6a, the particles are depleted in Cu (Fig. 6b) and mainly consist of Co (Fig. 6c). It was found by XRF that the observed CuBe2 alloy exhibits 0.2 wt.-% of Co, and EDS shows that Co is mostly concentrated in the found particles. Considering Fig. 6c, the Co-rich particles are randomly distributed and not accumulated at grain boundaries, leading to the assumption that these Co-rich particles do not influence the formation of a dendritic microstructure at the grain boundaries. The exact chemical composition of the Co-rich particles could not be detected with EDS, as the particles are smaller than 2 μm, which means that the electron beam also measures the area around the particles and distorts the result.

## Discussion

The deformed areas shown in Fig. 2a, b, which occur periodically with a distance of 50 mm, might be explained by a mechanical standing wave, which is induced by EMF. Such a standing wave consists of maxima with defined distances between them, where the maxima affect the inductor, the material is plastically deformed. This damage behavior weakens the Cu-inductor. However, the plastically deformed areas are not as critical as the observed cracks, pores, and blowholes in the CuBe2-inductor in Figs. 2c and 3. Such surface cracks were also found by Saadouki et al.^8^ in a fieldshaper made of a CuNiSi alloy that was used during EMF. In the fieldshaper, the first crack was initiated at the position where the highest stress level was reached. The large stress development was caused by the radial component of the Lorentz force. In addition, Scheffler^7^ also assumed that high current densities lead to large Lorentz forces that initiate cracks in the inductor or fieldshaper surface. The increased current density at the crack causes a temperature concentration. The resulting high temperature gradient leads to a high thermo-mechanical stress. In combination with the Lorentz force, the overall stress regime becomes electro-thermo-mechanical and causes local material failures. A further increase in the number of discharges, e.g., by an increase in the number of formed workpieces, intensifies the surface damages and leads to a damage evolution (see Fig. 3c). Pores form next to previously initiated cracks and grow with further electrical-thermo-mechanical loading during EMF, resulting in large pores or blowholes (see Fig. 3d). The formation of such damage is self-reinforcing, as the electric current induces stress at the crack tips^6,9–11^and the electrical-thermo-mechanical load is concentrated at existing cracks, leading to a high temperature gradient and thus to annealing, recrystallization, and localized melting at the crack tips due to Joule heating^7,10–12^. In^13^it was found that high current pulses are present at a pre-machined notch tip, resulting in the formation of a crack developing into a blowhole. The blowhole, in turn, causes blunting of the crack tip, thus retarding crack propagation. In addition, the EBSD measurements in Fig. 4 confirm that a fine-grained microstructure in the blowhole and at the grain boundaries is present due to melting and re-solidification processes during EMF. The electrical resistivity of metals is the sum of the temperature-dependent resistivity that results from the interaction of electrons and photons and the resistivity caused by lattice defects and impurities^14,15^. The higher electrical resistivity of grain boundaries leads to the concentration of thermal energy, resulting in local melting and re-solidification of material (Figs. 4a and 5a), which promotes the evolution of the damage as described above. In general, grain boundaries exhibit one to two orders of magnitude higher electrical resistivity than twin boundaries^16,17^. Kim et al.^17^ determined the electrical resistivity by a first-principles electronic structure method to (19.0–25.9) × 10^–12^ Ωcm^2^ at grain boundaries and (0.202–1.885) × 10^–12^ Ωcm^2^ at twin boundaries. As can be seen in Figs. 4a and 5b, no local melting and re-solidification was found at twin boundaries. Therefore, introducing twin boundaries is an appropriate procedure to increase the strength without sacrificing the electrical conductivity of copper alloys^18^. Twin boundaries can be induced by severe plastic deformation and subsequent heat treatment to directly tailor the microstructure. In addition, a relatively low recrystallization temperature should be used to increase the number of twins per grain, resulting in higher strength^19^. Furthermore, lattice defects hinder the movement of the electrons and therefore generally increase the electrical resistivity^20^. The electrical resistivity is also influenced by the size of the lattice defects. If the lattice defect is smaller than the mean free path of electrons, which is about 42 nm in copper alloys, the effect on the electrical resistivity is negligible^21^. This means that in addition to the hindering effect of grain boundaries, an increase in alloying element concentration^21^ and dislocation density, e.g., introduced by plastic deformation^22^reduces the electrical conductivity. In contrast, a high yield and ultimate tensile strength are favorable for inductor materials in order to withstand the severe mechanical load during EMF. Therefore, the more effective way to improve the strength without hindering the electron movement is to introduce small precipitates through (severe) plastic deformation and heat treatment in copper alloys^21,22^. As described earlier, the combination of a (severe) plastic deformation and subsequent heat treatment cannot only induce small precipitates but also twin boundaries, both known for their strengthening effect and low electrical resistivity, resulting in promising inductor materials for EMF.

## Conclusion

In summary, EMF enables the shaping of sheet metal with high strains and complex geometries. However, the damage behavior and damage evolution of copper alloy inductors used for EMF have been less studied so far. Therefore, this study aims to investigate the influence of different microstructural elements on the damage behavior of such EMF inductors made of copper alloy. The findings are summarized as:

1. EMF leads to plastically deformed areas on the inductor’s surface of copper alloys with periodic distance in between, leading to the assumption that a mechanical standing wave is induced by EMF.
2. After several discharges, cracks appear in the deformed areas. In addition to the electrical and mechanical load, the thermal load during EMF is concentrated at microstructural elements with a high electrical resistivity, such as grain boundaries, resulting in melting and re-solidification processes there. Due to high Lorentz forces, cracks propagate through the grain boundaries, forming small pores at them.
3. The damage process is self-reinforcing as the electrical-thermo-mechanical load is concentrated at the cracks, which form larger pores or blowholes after several discharges, thus damaging the inductor.
4. The influence of Co-rich particles on the damage behavior of the investigated CuBe2 alloy seems to be negligible, as they are not concentrated at grain boundaries, which are the origin of the damage evolution.

The next step is to increase the strength without sacrificing the electrical conductivity to enhance the lifetime of the inductor. Therefore, severe plastic deformation and a subsequent heat treatment will be used to increase the number of twin boundaries and to induce small precipitates, which both hinder the movement of dislocations but not that of electrons, leading to a high strength and high electrical conductivity. At this point, the damage behavior of copper inductors is described, and first explanations are given. However, the stepwise damage evolution is not fully understood right now. Therefore, the microstructure should be investigated after a selected number of discharges. The gained knowledge is valuable, not only to understand the damage behavior of a copper inductor during EMF but also for further applications with high electrical and mechanical loads.

## Data Availability

Data sets generated during the current study are available from the corresponding author on reasonable request.
